# From age to frailty: redefining chronic pain characterization

**DOI:** 10.1007/s40520-025-03273-4

**Published:** 2025-12-02

**Authors:** Pablo Mourelle-Sanmartín, Laura Lorenzo-López, José Carlos Millán-Calenti, Melissa Kathryn Andrew, Olga Theou

**Affiliations:** 1 Geriatris Department, University Hospital Lucus Augusti, Lugo, Galicia Spain; 2 Gerontology and Geriatrics Research Group, Instituto de Investigación Biomédica de A Coruña (INIBIC), Universidade da Coruña, Complexo Hospitalario Universitario de A Coruña (CHUAC), SERGAS, A Coruña, Spain; 3 Department of Medicine, Dalhousie University, Halifax, NS Canada; 4 School of Physiotherapy, Dalhousie University, Halifax, NS Canada

**Keywords:** Frailty, Biological age, Chronic pain, Pain characteristics, Pain assessment

## Abstract

**Background:**

Chronic pain in older adults is highly prevalent, multifactorial, and often associated with greater intensity, multisite involvement, and functional impairment. Despite its burden, it remains frequently underdiagnosed and undertreated. Chronological age alone does not adequately capture biological vulnerability or interindividual variability in pain expression.

**Aims:**

To examine the associations of frailty, an indirect marker of biological age, and chronological age with chronic pain characteristics.

**Methods:**

We conducted a cross-sectional study including 455 adults (≥18 years) recruited from primary care. Thirty-three pain characteristics were assessed through structured interviews. Frailty was quantified using a 31-item Frailty Index based on the deficit accumulation model. Associations of frailty, chronological age, and sex with each pain variable were analyzed using multivariable linear and logistic regression models.

**Results:**

Most pain characteristics were more consistently associated with frailty than with chronological age, although effect sizes were modest (sr^2^ typically 1–5%). Frailty correlated with greater pain intensity (sr 0.23, r^2^ 5.3%), higher frequency (sr 0.10, r^2^ 1.1%), and continuous or mixed-type pain (OR 0.97, 95% CI 0.95–0.99). In contrast, chronological age primarily predicted temporal aspects, including pain duration, diagnostic delay, and time to first analgesic prescription. Age and frailty showed opposite directions of association for certain domains, such as accompanying symptoms and daily pain duration.

**Conclusion:**

Frailty provides complementary information to chronological age in characterizing chronic pain. Integrating frailty assessment into routine pain evaluation may enable more individualized management, enhance pain control, and reduce age-related disparities in clinical care.

**Supplementary Information:**

The online version contains supplementary material available at 10.1007/s40520-025-03273-4.

## Key points

Frailty, as a proxy for biological age, showed more consistent associations than chronological age with several chronic pain characteristics, including intensity, mixed-type pain, continuous course, and accompanying symptoms.

Integrating frailty assessment into pain evaluation offers a biologically grounded and individualized framework, particularly relevant for aging populations.

**Supplementary Information:**

The online version contains supplementary material available at 10.1007/s40520-025-03273-4.

## Introduction

Chronic pain, as defined by the International Association for the Study of Pain (IASP), is pain persisting for ≥3 months and recurring on at least three days per week [1]. It is a complex, multifactorial condition encompassing physical, psychological, and social dimensions. In older adults, it often presents as mixed-type pain with a marked tendency toward chronicity [2–4]. Chronic pain exerts bidirectional effects on health, being associated with greater comorbidity burden, functional and cognitive decline, depression, sleep disturbances, reduced social participation, and poorer quality of life [2, 5]. These consequences are amplified in later life, where chronic pain frequently evolves into high-impact chronic pain, particularly among frail individuals [6, 7]. Despite its prevalence, chronic pain in older adults remains underdiagnosed and undertreated [8].

With advancing age, chronic pain tends to affect multiple anatomical sites–most frequently the back (5–45%), neck (20%), hips (20%), knees (18%), hands (15%), and feet (14%)–while head pain, typical of acute conditions, becomes less common (3–4%) [2]. Multisite pain is clinically relevant in older adults, as it is associated with functional decline, disability, and falls, and has been proposed as a potential geriatric syndrome [5, 9, 10]. Pain intensity generally increases with age, although differences between older and middle-aged adults are less pronounced [2].

Frailty represents a state of increased vulnerability to stressors, reflecting diminished physiological reserve and higher risk of adverse outcomes such as hospitalization, functional decline, and mortality [11]. Two main frameworks are commonly used. The frailty phenotype conceptualizes frailty as a biological syndrome of functional decline, accelerated by disease, medication, or aging. Operationalized through five physical criteria–unintentional weight loss, weakness, slowness, exhaustion, and low physical activity–it classifies individuals as robust, prefrail, or frail. Its primary advantage is its simplicity and rapid application, supporting widespread use in epidemiological and clinical settings. However, it captures almost exclusively physical attributes and does not incorporate comorbidity, mental health, or social dimensions [12, 13]. In contrast, the deficit accumulation model defines frailty as a multidimensional risk state resulting from the accumulation of health deficits (symptoms, diseases, disabilities, and laboratory abnormalities) across four domains: system reserve (comorbidities), mental health (particularly cognition), physical function, and social aspects. It is operationalized through a frailty index (FI), calculated as the proportion of deficits present relative to the total considered, yielding a continuous score (0–1) that captures subtle gradients of vulnerability [14, 15].

Frailty indices serve not as an end in themselves but as instruments to quantify individual vulnerability. Consensus guidelines recommend that a robust FI include at least 30 items across multiple domains and demonstrate a positive, non-redundant correlation with chronological age [16]. FIs have been validated from early adulthood (18 years) onwards and applied across diverse disciplines, including cardiology and population-based research, with methodological recommendations supporting their use below age 65. The FI, although more resource-intensive, sensitively captures systemic vulnerability across the life course and is increasingly advocated beyond geriatric practice [15, 17–20]. Crucially, it has been validated as a proxy for biological age, enabling estimation of biological age above or below chronological age. This distinction allows exploration of discrepancies between biological and chronological age, helping counter ageist assumptions and reinforcing the clinical relevance of frailty. Within this framework, frailty level reflects an individual´s position on the continuum of accumulated deficits, vulnerability denotes the broader multidimensional risk state, and biological age corresponds to the FI-derived estimate [21, 22]. Both approaches predict adverse outcomes: the phenotype is simpler and widely used in biological research, while the FI provides stronger age-related trajectories and mortality associations, albeit less feasible in fast-paced settings. Ultimately, the choice between them should be guided by the specific clinical or research objective [23, 24].

Frailty shows a bidirectional, dose-dependent relationship with chronic pain, regardless of whether measured using phenotypic or deficit accumulation models [25, 26]. However, studies specifically addressing how chronic pain characteristics relate to frailty level and chronological age remain scarce and methodologically heterogeneous. Most have focused on pain intensity or the number of painful sites, typically using the frailty phenotype [4, 5, 27–47], the Frailty Index [37, 48–55], or alternative tools [56, 57], with limited evaluation of the multidimensional expression of pain. Importantly, these studies rarely consider biological age, despite the fact that frailty has been validated as its proxy, and seldom contrast it directly with chronological age in the characterization of chronic pain.

Whether frailty provides a more robust explanation of variability in chronic pain characteristics than chronological age remains uncertain. To address this gap, we examined the associations of frailty–an indirect estimate of biological age–and chronological age with a comprehensive set of chronic pain characteristics. Our goal was to clarify their relative contributions across the adult lifespan and highlight the value of frailty as a proxy for biological age in chronic pain research.

## Methods

### Study design and participants

We conducted an analytical cross-sectional study involving community-dwelling adults with chronic pain. Participants were recruited from two primary care centers in Lugo province (Galicia, Spain) during a three-month period. Inclusion criteria were: [1] age≥18 years [2], fulfillment of IASP criteria for chronic pain (pain persisting for ≥3 months and occurring ≥3 days per week), and [3] voluntary participation with informed consent. Exclusion criteria were: (a) refusal to participate, (b) cognitive impairment precluding reliable data collection–defined as Global Deterioration Scale (GDS) score ≥5 or caregiver-confirmed unreliability–, and (c) inability to complete the structured interview. Residents in long-term care facilities were not systematically excluded and were therefore represented in the analytical sample (*n* = 37, 8.1%; see Appendix 1).

Sample size was estimated to ensure robust prevalence estimation and adequate power for multivariable analyses. Based on reported chronic pain prevalence ranging from 11% to 50%, a conservative estimate of 50% was adopted, with 5% precision and 95% confidence, yielding a minimum required sample size of 384. This was increased to 455 to account for potential inconsistencies and ensure sufficient power for secondary analyses. For instance, assuming a 20% prevalence for a binary outcome, the final sample provided ≥90 events–enough to support up to six predictors in logistic regression (15 events per variable). Post hoc power calculations for linear regression (three predictors, f^2^ = 0.05, α = 0.05, power = 0.80) indicated a required sample size of 160, confirming the adequacy of the final cohort.

A consecutive recruitment strategy was implemented, whereby all individuals attending routine primary care visits were systematically screened for chronic pain according to IASP criteria. This pragmatic approach enabled broad inclusion of eligible participants, although it was inherently limited to the clinic-attending population rather than a randomly selected community cohort. Of 1646 individuals assessed, 713 met the IASP criteria for chronic pain. Among them, 455 completed the structured evaluation and comprised the final study cohort. The remaining 258 were not interviewed due to logistical constraints (*n* = 50), refusal (*n* = 2), or repeated visits during the recruitment period resulting in duplication (*n* = 206). These cases were included in the overall prevalence analysis but excluded from this dataset, which focused on detailed characterization through structured interviews. Trained researchers conducted 20–30 min interviews, collecting 169 variables across multiple domains: demographics (*n* = 2), chronic conditions (*n* = 34), pain features (*n* = 121), self-rated health (*n* = 1), physical function and disability (*n* = 4), mental health (*n* = 5), and social health (*n* = 1). All data were corroborated using electronic health records.

The study protocol was approved by the Santiago-Lugo Research Ethics Committee and classified by the Spanish Agency for Medicines and Medical Devices (AEMPS) (LAO-PAR-2018-01).

### Pain characteristics

A total of 33 variables capturing chronic pain characteristics were analyzed and grouped into five domains: [1] pain duration (total time with pain, time to etiological diagnosis, time to first treatment [pharmacological, non-pharmacological, or both], and time to pain control) [2], pain location (number and anatomical distribution, both at onset and current) [3], pain-modifying factors (movement, temperature, and medication) [4], accompanying symptoms (nausea, asthenia, anorexia, and sleep disturbances), and [5] other characteristics: frequency (days per week), daily duration (hours), intensity (Visual Analogue Scale 0–10), predominant type (mechanical, neuropathic, or mixed), pain course (intermittent or continuous), and social limitation due to pain (SF-12 questionnaire).

### Frailty assessment: operationalizing a frailty index

Frailty was assessed using the deficit accumulation approach to capture the continuum of vulnerability and provide an indirect estimate of biological age. A 31-item Frailty Index (FI) was constructed following standard deficit accumulation procedures [16].

Of the 169 collected variables, 127 were excluded because they did not represent specific health deficits. Eligible variables were coded from 0 (no deficit) to 1 (full deficit). Ordinal responses were scaled proportionally, and continuous variables were normalized to the 99th percentile. Variables with extremely low prevalence (< 1%) or lacking association with age were excluded, except for “rheumatoid arthritis” and “other arthritis”, which were retained due to clinical relevance. Inter-variable correlations were examined to minimize redundancy.

The final FI included 31 variables distributed across five domains: chronic conditions (*n* = 22), mental health (*n* = 4), physical function (*n* = 3), social health (*n* = 1), and self-rated health (*n* = 1) (see Appendix 2). Each participant´s FI score was calculated as the proportion of accumulated deficits (range 0 to 1). For descriptive purposes, participants were categorized into five frailty levels: non-frail (0-0.1), very mildly frail (0.11–0.2), mildly frail (0.21–0.3), moderately frail (0.31–0.4), and severely frail (≥0.41).

In this cohort, FI scores ranged from 0.04 to 0.62 (mean 0.28±0.13), displaying the expected right-skewed distribution (see Appendix 3). These findings align with previous reports in both clinical and population-based samples, supporting the external validity of the index. No sex-based differences in FI scores were observed, consistent with prior literature [15, 16, 23]. Overall, these results confirm the methodological robustness of the FI and its suitability for assessing frailty across the adult lifespan. This validated index was subsequently used as the primary operational measure of frailty in all analyses.

### Statistical analysis

Descriptive statistics were performed for all variables. Continuous variables were summarized as means, standard deviations (SD), and 95% confidence intervals (CI). Categorical variables were expressed as frequencies and percentages. Normality and distributional assumptions were verified visually and statistically.

Bivariate associations between the FI and pain characteristics were examined using Pearson or Spearman correlation coefficients, as appropriate. Key associations were visualized graphically.

Multivariable linear regression models were constructed for continuous pain outcomes, with FI score, age, and sex as independent variables. We report unstandardized coefficients (B), 95% CI, and semipartial correlation coefficients (sr^2^) to quantify unique variance explained. Logistic regression was used for binary outcomes, with results expressed as odds ratios (OR) and 95% CIs. Outcomes with fewer than 30 events (< 7% prevalence) were excluded from multivariable models to avoid unstable estimates.

Model assumptions were assessed using residual plots and diagnostic statistics. Multicollinearity was evaluated via variance inflation factors (VIF), with no issues identified. As no missing data were present, imputation was not required. All analyses were conducted using IBM^®^SPSS^®^ Statistics version 28.0.1.1.

## Results

### Participant characteristics

A total of 455 participants were included (mean age 63.3±1.8 years; range: 20–103), of whom 46.2% were aged ≥65 years. Based on the FI, 19.8% met criteria for severe frailty (FI≥0.41). Sociodemographic and clinical characteristics by frailty level are summarized in Table 1.

**Table 1:** Main pain characteristics of participants with chronic pain stratified by frailty levels (n, % [95% CI], or mean±SD [range])

		Non-frail (0-0.10)	Very mildly frail (0.11–0.20)	Mildly frail (0.21–0.30)	Moderately frail (0.31–0.40)	Severely frail (≥0.41)
Prevalence		53, 11.7% (8.9–14.8)	94, 20.7% (17.1–24.6)	142, 31.1% (27.1–35.6)	76, 16.7% (13.5–20.3)	90, 19.8% (16.3–23.6)
Sex	Female	28, 52.8% (39.5–65.8)	59, 62.8% (52.7–72.0)	84, 59.2% (51.0–67.0)	57, 75.0% (64.5–83.7)	46, 51.1% (40.9–61.3)
	Male	25, 47.2% (34.2–60.5)	35, 37.2% (28.0-47.3)	58, 40.8% (33.0–49.0)	19, 25.0% (16.3–35.5)	44, 48.9% (38.7–59.1)
Age	Overall years	44.35 ± 2.84 (26–68)	51.85 ± 3.55 (29–103)	64.18 ± 2.75 (20–94)	68.65 ± 3.16 (36–92)	80.17 ± 2.76 (50–96)
	18–40 years	17, 32.1% (20.7–45.3)	32, 34.0% (25.1–44.0)	14, 9.9% (5.8–15.6)	3, 3.5% (1.1–10.2)	0
	41–64 years	33, 62.3% (48.8–74.4)	48, 51.1% (41.1–61.0)	56, 39.4% (31.7–47.6)	26, 34.4% (24.3–45.3)	16, 17.8% (11.0-26.6)
	65–79 years	3, 5.6% (1.6–14.3)	5, 5.3% (2.1–11.3)	49, 34.5% (27.1–42.6)	33, 43.5% (32.7–54.6)	14, 15.6% (9.2–24.1)
	80–89 years	0	3, 3.2% (0.9–8.3)	20, 14.1% (9.1–20.5)	13, 17.3% (9.9–26.7)	43, 47.8% (37.7–58.0)
	> 90 years	0	6, 6.4% (2.7–12.7)	3, 2.1% (0.6–5.5)	1, 1.3% (0.1-6.0)	17, 18.8% (11.9–27.9)
Charlson comorbidity		62.59 ± 1.173 (24–98)	43.39 ± 1.97 (14–98)	37.44 ± 1.71 (2–75)	23.42 ± 0.70 (0–30)	13.28 ± 0.47 (0–20)
Months since pain onset		54.20±3.86 (3-168)	147.00±17.66 (3-756)	214.23±18.22 (3-780)	244.87±13.72 (3-588)	391.23±16.60 (48–756)
Current pain locations number		1.82±0.07 (1–4)	2.37±0.09 (1–5)	3.09±0.12 (1–6)	3.46±0.16 (1–8)	4.97±0.21 (1–10)
Frequency of pain (days/week)		6.65±0.07 (4–7)	6.86±0.07 (4–7)	6.86±0.05 (5–7)	6.96±0.05 (5–7)	7.00±0.00 (7–7)
Duration of pain per day (hours)		14.93±0.52 (2–24)	16.39±0.45 (4–24)	16.84±0.38 (8–24)	16.96±0.38(8–24)	16.97±0.28 (12–24)
Intensity of pain		5.37±0.12 (3–8)	5.55±0.19 (0–8)	6.63±0.16 (3–10)	6.74±0.14 (4–10)	6.97±0.09 (5–9)
Mechanical pain type		32, 60.4% (46.9–72.7)	41, 43.6% (33.9–53.7)	59, 41.5% (33.7–49.8)	24, 31.6% (22.0-42.6)	27, 30.0% (21.3–40.0)
Neuropathic pain type		11, 20.8% (11.6–33.0)	28, 29.8% (21.3–39.5)	43, 30.3% (23.2–38.2)	14, 18.4% (11.0-28.2)	17, 18.9% (11.9–27.9)
Mixed pain type		10, 18.9% (10.1–30.9)	26, 27.7% (19.4–37.3)	41, 28.9% (21.9–36.7)	37, 48.7% (37.7–59.8)	45, 50.0% (39.8–60.2)
Intermittent pain course		40, 75.5% (62.8–85.5)	66, 70.2% (60.5–78.7)	106, 74.6% (67.0-81.3)	51, 67.1% (56.1–76.9)	50, 55.6% (45.2–65.5)
Continuous pain course		13, 24.5% (14.5–37.2)	28, 29.8% (21.3–39.5)	36, 25.4% (18.7–33.0)	25, 32.9% (23.1–43.9)	40, 44.4% (34.5–54.8)

Percentages were calculated based on the number of participants within each frailty level

Higher FI scores were associated with longer pain duration, greater intensity and frequency, and a higher number of pain locations. Compared with non-frail participants, the most frail group had approximately sevenfold longer pain duration and nearly three times as many painful sites. The prevalence of mixed-type and continuous pain increased progressively with frailty, whereas mechanical and intermittent patterns became less frequent.

### Associations of frailty, chronological age, and sex with chronic pain characteristics

**Fig. 1:**
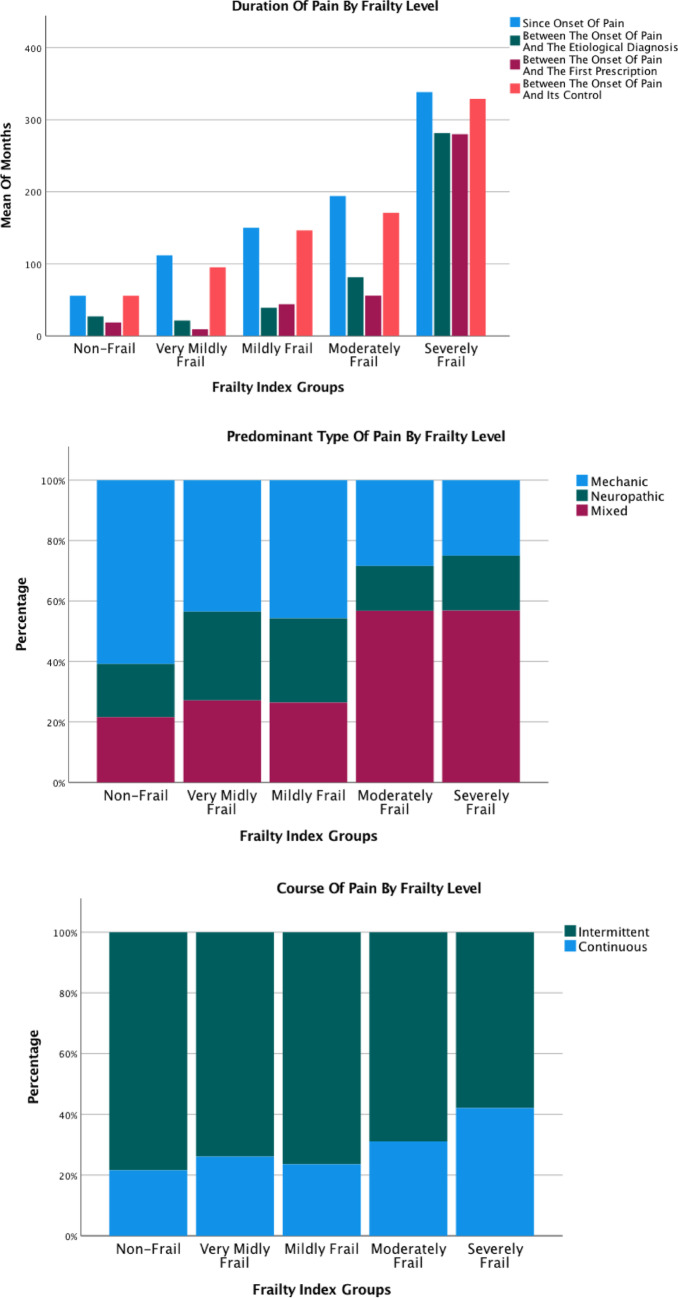
Main pain characteristics across frailty levels. Only some main pain characteristics are shown. For more information see Appendix 3

Most pain characteristics were positively correlated with frailty (Table 2; Fig. 1, Appendix 4). Chronological age showed stronger associations than frailty for certain temporal dimensions, including total time with pain (sr 0.42, r^2^ 17.8%), time to etiological diagnosis (sr 0.40, r^2^ 16.2%), and time to first analgesic prescription (sr 0.33, r^2^ 11.0%). Conversely, frailty correlated more closely with time to pain control (sr 0.27, r^2^ 7.0%) and to the number of painful locations (sr 0.29, r^2^ 8.6%). Frailty also showed consistent, though modest, associations with pain-modifying factors such as movement, medication, and temperature (sr 0.19, r^2^ 3.7%).

**Table 2:** Association between chronic pain characteristics and frailty

Domain	Pain characteristics	Correlation coefficient (*p*-value) with frailty	
**Duration of pain (months)**	Since the onset of pain	**0.513**	**< 0.001**
	Between the onset of pain and the etiological diagnosis	**0.416**	**< 0.001**
	Between the onset of pain and the first prescription (pharmacological and non-pharmacological)	**0.448**	**< 0.001**
	Between the onset of pain and its control	**0.463**	**< 0.001**
**Pain locations**	Number of pain locations at onset of pain	**0.122**	**0.010**
	Number of current pain locations	**0.520**	**< 0.001**
	Cervical location (current)	0.161*	**< 0.001**
	Dorsal location (current)	0.053*	0.264
	Lumbar location (current)	0.287*	**< 0.001**
	Totalgia location (current)	0.124*	**0.009**
	One shoulder location (current)	0.038*	0.422
	Both shoulders location (current)	0.125*	**0.008**
	One hip location (current)	0.314*	**< 0.001**
	Both hips location (current)	0.269*	**< 0.001**
	One knee location (current)	0.244*	**< 0.001**
	Both knees location (current)	0.269*	**< 0.001**
	Headache location (current)	0.158*	**< 0.001**
	Others location (current)	0.065*	0.169
**Factors affecting pain**	Number of factors affecting pain	**0.353**	**< 0.001**
	Movement affecting pain	**0.217**	**< 0.001**
	Temperature affecting pain	0.270	0.264
	Medication affecting pain	**0.255**	**< 0.001**
**Accompanying symptoms**	Number of pain accompanying symptoms	**0.332**	**< 0.001**
	Nausea as a pain accompanying symptom	0.053*	0.265
	Asthenia as a pain accompanying symptom	0.316*	**< 0.001**
	Anorexia as a pain accompanying symptom	0.276*	**< 0.001**
	Sleep disturbance as a pain accompanying symptom	0.199*	**< 0.001**
**Other characteristics**	Frequency of pain (days/week)	**0.164**	**< 0.001**
	Duration of pain per day (hours)	**0.123**	**0.010**
	Intensity of pain	**0.372**	**< 0.001**
	Type of pain (mechanical, neuropathic, mixed)	**0.278**	**< 0.001**
	Course of pain (intermittent, continuous)	**0.228**	**< 0.001**
	Social limitation due to pain	**-0.296**	**< 0.001**

*Spearman correlation coefficient. Otherwise Pearson correlation coefficient

**Bolded values** imply statistical significance (*p* < 0.05)

Regarding anatomical distribution, frailty was significantly associated with dorsal (OR 1.03, 95% CI 1.01–1.06) and lumbar pain (OR 1.05, 95% CI 1.02–1.07), whereas sex–rather than age or frailty–was the primary determinant of pain distribution at onset (sr 0.25, r^2^ 6.3%).

Frailty was also significantly related to several accompanying symptoms, including asthenia (OR 1.10, 95% CI 1.07–1.13), anorexia (OR 1.07, 95% CI 1.05–1.10), and sleep disturbance (OR 1.09, 95% CI 1.07–1.12). Chronological age showed an inverse association with these symptoms (sr -0.33, r^2^ 11.1%).

Pain intensity correlated modestly but consistently with frailty (sr 0.23, r^2^ 5.3%), while chronological age showed no significant association. Similar patterns were observed for pain frequency (FI: sr 0.10, r^2^ 1.1%; sex: sr 0.18, r^2^ 3.2%) and daily pain duration (FI: sr 0.18, r^2^ 3.1%; age: sr -0.14, r^2^ 1.9%). Pain course was associated with frailty (continuous pain: OR 0.97, 95% CI 0.95–0.99) but not with age.

Both frailty and chronological age influenced mixed-type pain and social limitation due to pain. Notably, social limitation showed an inverse association with frailty (OR 0.92, 95% CI 0.90–0.94), possibly reflecting lower baseline social participation among frailer individuals or the contribution of non-pain-related limiting factors.

**Fig. 2:**
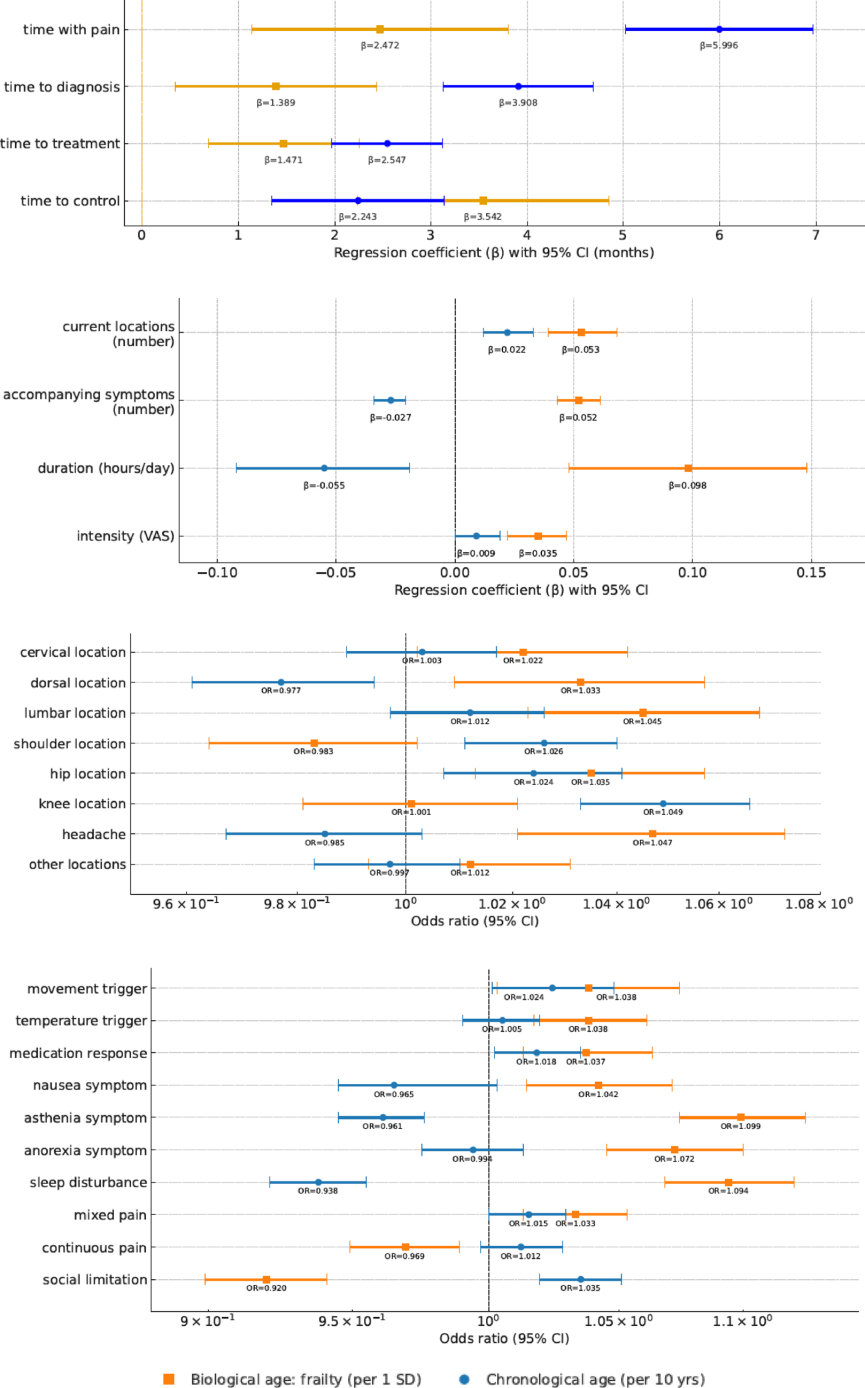
Associations of chronological and biological age with pain-related characteristics. Forest plots showing the associations between chronological age (●, per 10 years) and biological age (■, frailty per 1 SD) with pain-related variables. Panels A–B display regression coefficients (β, 95% CI) from linear models for continuous outcomes, whereas Panels C–D show odds ratios (OR, 95% CI; logarithmic scale) from logistic models for categorical outcomes. In Panel B, three variables were excluded due to non-significance or substantial overlap between coefficients: number of onset locations, number of pain-modifying factors, and pain frequency. In Panels C–D, total number of pain locations, both shoulders, and both knees were excluded because of non-significance or nearly identical OR values. Full regression outputs are provided in Supplementary Appendix 5. VAS = visual analog scale

Table 3; and Fig. 2 summarize the main multivariable associations between biological age (estimated by the FI) and chronological age with key pain-related characteristics. Full regression models–including sex as covariate and all pain variables– are presented in Appendix 5. Variables with fewer than 30 events (< 7% prevalence) were excluded from multivariable analyses to avoid unstable estimates.

**Table 3:** Association of frailty (FI) and age with chronic pain characteristics

Panel A. Continuous outcomes (linear regression models)				
Outcome	Predictor	β (95% CI)	*p*-value	sr^2^ (%)
Time to pain control	FI (per 1SD)	3.54 (2.24–4.85)	**< 0.001**	0.27 (7.0%)
	Age (per 10 yrs)	2.24 (1.35–3.14)	**< 0.001**	0.24 (6.0%)
Pain locations	FI (per 1 SD)	0.05 (0.04–0.07)	**< 0.001**	0.29 (8.6%)
	Age (per 10 yrs)	0.02 (0.01–0.03)	**< 0.001**	0.17 (2.7%)
Accompanying symptoms (number)	FI (per 1SD)	0.05 (0.04–0.06)	**< 0.001**	0.46 (21.4%)
	Age (per 10 yrs)	-0.03 (-0.03 to -0.02)	**< 0.001**	-0.33 (11.1%)
Duration of pain (h/day)	FI (per 1SD)	0.10 (0.05–0.15)	**< 0.001**	0.18 (3.1%)
	Age (per 10 yrs)	-0.06 (-0.09 to -0.02)	**0.003**	-0.14 (1.9%)
Intensity of pain	FI (per 1SD)	0.04 (0.02–0.05)	**< 0.001**	0.23 (5.3%)
	Age (per 10 yrs)	0.01 (0.001–0.02)	**0.048**	0.08 (0.7%)

Panel B. Categorical outcomes (logistic regression models)				
Outcome	Predictor	OR (95% CI)	*p*-value	*N* events
Lumbar pain location	FI (per 1SD)	1.05 (1.02–1.07)	**< 0.001**	291 (65.4%)
	Age (per 10 yrs)	1.01 (0.99–1.03)	0.103	291 (65.4%)
Medication modifying pain	FI (per 1 SD)	1.04 (1.01–1.06)	**0.003**	338 (76.0%)
	Age (per 10 yrs)	1.02 (1.00-1.04)	**0.031**	338 (76.0%)
Sleep disturbance	FI (per 1SD)	1.09 (1.07–1.12)	**< 0.001**	266 (59.8%)
	Age (per 10 yrs)	0.94 (0.92–0.96)	**< 0.001**	266 (59.8%)
Mixed pain	FI (per 1SD)	1.03 (1.01–1.05)	**0.001**	154 (43.6%)
	Age (per 10 yrs)	1.02 (1.00-1.03)	**0.041**	154 (43.6%)
Continuous curse of pain	FI (per 1SD)	0.97 (0.95–0.99)	**0.003**	137 (30.8%)
	Age (per 10 yrs)	1.01 (0.99–1.03)	0.127	137 (30.8%)

All models were adjusted for sex; coefficients for sex are presented in Appendix 3.

**Bolded values** indicate statistical significance (*p* < 0.05).

Regression coefficients for frailty are expressed per one standard deviation (1 SD) increase in the Frailty Index; age coefficients are expressed per 10-year increase in chronological age.

## Discussion

Frailty level was consistently associated with most pain characteristics, aligning with previous–though limited–studies linking frailty to selected pain domains. Beyond corroborating these findings, this study offers novel insights into specific pain dimensions that had not been systematically examined in relation to frailty. In some domains–such as dorsal pain and movement-modifying factors–the strength of association for frailty was comparable to that of age and sex. In others–including pain frequency, intensity, cervical and lumbar pain, and pain course–chronological aging was not statistically significant or even showed an opposite direction of association compared to frailty. These results suggest that, among individuals with similar frailty levels, older adults may experience fewer accompanying symptoms and shorter daily pain duration. Although statistically robust, most effect sizes were modest (sr^2^ typically 1–5%), indicating that frailty explains only part of chronic pain variability. This highlights the multifactorial nature of pain, while underscoring the incremental value of frailty as a multidimensional marker of vulnerability that complements chronological age. Interpreting pain solely through the lens of age–without accounting for frailty–risks diagnostic delays, suboptimal management, and ageist assumptions in clinical care.

Chronological age nonetheless emerged as the main predictor of delayed etiological diagnosis and treatment initiation, pointing toward potential manifestations of ageism within healthcare. Such delays likely reflect multilevel barriers [8]: (a) patient-level factors (e.g., beliefs that pain is a normal part of aging, fear of addiction, or reluctance to appear weak); (b) clinician-level (e.g., limited geriatric training, lack of frailty-adapted protocols, implicit biases); and (c) system-level (e.g., fragmented care and accessibility barriers). Addressing these challenges requires integrating geriatric principles into pain care, developing frailty-sensitive clinical guidelines, and promoting health system reforms to reduce age-related disparities.

Previous studies, such as Rodríguez-Sánchez I et al. (2019), identified a positive relationship between frailty and the number of painful sites, typically using dichotomous classifications (localized vs. widespread pain) [5, 37, 48, 51]. Our findings expand on this evidence, identifying frailty as the most consistent correlate of the accumulation of painful sites over time, even though absolute effect sizes remained moderate.

The literature on anatomical pain distribution remains heterogeneous and, at times, contradictory. Many studies focused on isolated body regions or pathologies, assessed partial frailty constructs, or failed to control for confounders [4, 27–29, 34, 35, 39, 40, 43, 56, 57]. By examining multiple pain locations simultaneously while adjusting for age and sex, our study provides a more integrated perspective. For example, while Tsuji et al. (2020) reported associations between lumbar pain, Fried frailty, and age, our results identified the Frailty Index as the only significant predictor. Similarly, Bindawas et al. (2018) linked knee pain to frailty using the FRAIL scale; we found that unilateral knee pain was more strongly associated with age, whereas bilateral pain was predominantly related to frailty. This pattern extended to other locations such as hips and shoulders. Bilateral pain tended to reflect frailty, while unilateral pain was linked to chronological age. These findings may reflect underlying etiologies: focal pain from trauma or localized inflammation is more common in younger adults, whereas degenerative or systemic inflammatory processes–often related to biological aging–are more strongly associated with frailty. Assessing pain exclusively by chronological age risks overlooking treatable conditions, whereas integrating biological age through frailty assessment can facilitate earlier, more targeted interventions.

Pain intensity has been the most extensively studied dimension of pain in relation to frailty. Consistent with prior evidence [4, 30–33, 35, 36, 38, 42, 44–50, 52–57], frailty was positively associated with pain intensity across multiple domains, with female sex also exerting a significant influence. These associations likely reflect both the structural burden of disease and psychosocial amplification through factors such as sleep disturbance, depression, or cognitive decline–conditions more prevalent among women and individuals with higher frailty level. Conversely, chronological age was not a significant predictor of pain intensity, diverging from Shega et al. (2013) but consistent with hypotheses that older adults may develop adaptive coping mechanisms over time. Such adaptation might partly explain the paradoxically lower mortality reported among some older individuals with chronic pain compared with those without pain [53].

The social dimension of pain also warrants attention. While previous studies reported inconsistent associations between frailty and pain-related social limitation [33, 52, 55, 56], our results revealed an inverse relationship. This may reflect contextual factors: older adults in our setting may have lower baseline social participation [58], reducing the perceived impact of pain on social functioning. It is also possible that individuals with advanced frailty levels adapt by prioritizing essential interactions or that social limitations arise from comorbid or functional factors rather than pain itself. These results highlight the need to address subjective loneliness, sociocultural context, and rural integration when evaluating the social consequences of pain.

To our knowledge, this is the first study to jointly examine frailty associations with pain modifiers, accompanying symptoms, frequency, duration, type, and course. Frailty correlated with both continuous and mixed-type pain, suggesting stronger contributions from mental component and the cumulative burden of tissue-damaging conditions. Although Ardoino et al. (2020) described frailty associations with somatic or visceral pain compared with neuropathic pain, they did not explore predominant pain types in detail. Our finding that pain frequency increased with frailty levels aligns with Rodríguez-Sánchez et al. (2019) but offers greater resolution by analyzing frequency as a continuous rather than categorical variable.

The main limitation of this study is its cross-sectional design, which inherently precludes causal inference. Accordingly, only associations between frailty and chronic pain characteristics can be described, without establishing directionality (e.g., whether frailty predisposes to specific pain features or vice versa). This limitation is particularly relevant for outcomes such as mortality, where longitudinal follow-up would be required to establish causal pathways.

Regarding the recruitment strategy, several aspects merit consideration. In terms of internal validity, consecutive sampling minimized selection bias within the primary care population, and the use of standardized IASP criteria ensured diagnostic consistency. The structured assessment, conducted by trained researchers, and the inclusion of institutionalized participants strengthened the robustness of case identification and internal comparisons. Nonetheless, the exclusion of participants with advanced cognitive impairment (GDS≥5) may have indirectly limited representation of the most severely frail individuals.

With respect to external validity, the study reflects a realistic Southern European primary care context. However, its restriction to two centers in a single Spanish province may limit generalizability to broader populations or healthcare settings. In this regard, future studies including larger and more diverse international samples are warranted to confirm and extend these results. In contrast, the inclusion of both middle-aged and older adults, as well as community-dwelling and institutionalized individuals, enhances clinical applicability across the frailty continuum. Nevertheless, extrapolation to randomly selected community samples or to non-European healthcare systems should be made cautiously. Although consecutive recruitment minimized bias within the clinical population, residual selection bias cannot be completely excluded. Overall, the study design ensured internal consistency, although its external representativeness remains context-dependent.

Covariates such as comorbidity burden, medication use, and psychosocial domains were collected but not entered individually into regression models, as they constitute integral components of the FI. Including them separately would have introduced redundancy and collinearity, limiting interpretability. This analytic choice reinforces the validity of the FI as a multidimensional construct of vulnerability, but it precludes disentangling the independent effects of specific domains.

Finally, although associations between frailty and pain characteristics were consistent across models, effect sizes were generally modest (sr^2^ typically 1–5%), reflecting the multifactorial nature of chronic pain and emphasizing that frailty is one–though clinically relevant–dimension within this complex interplay.

Despite these limitations, the study has several strengths. Chronic pain was defined according to IASP standards, ensuring methodological rigor and comparability with international research. Pain was assessed comprehensively across multiple domains, providing a multidimensional characterization rarely achieved in population-based samples. The cohort spanned the full adult age range (≥ 18 years), with no upper limit, thus capturing age-related variability across the lifespan. Institutionalized participants were not systematically excluded and represented 8.1% of the analytical sample (Appendix 1), broadening applicability to vulnerable populations.

A further strength lies in the operationalization of frailty using a deficit accumulation–based Frailty Index, applied consistently across all ages. This approach conceptualizes frailty not merely as a sum of comorbidities but as a multidimensional proxy of biological age and global vulnerability. By analyzing both chronological and biological age (frailty) while adjusting for sex–a known determinant of pain expression–this study offers a nuanced perspective on the interplay between aging and pain. This multidimensional framework provides a more clinically meaningful lens for understanding chronic pain across the adult life course.

Complete data availability and a relatively large sample size further reinforce the robustness and reliability of our analyses. Our findings align with previous literature linking frailty to pain characteristics but extend this knowledge by demonstrating these associations in a general primary care cohort, including institutionalized patients. This broad scope enhances external validity and underscores the added value of frailty-informed perspectives in real-world care.

Future longitudinal, multicenter studies conducted across diverse healthcare systems and age ranges are warranted to clarify causal pathways, temporal dynamics, and domain-specific contributions. Future guidelines for chronic pain management in older adults should incorporate frailty-informed frameworks, emphasizing their complementarity–rather than substitution–for chronological age in clinical decision-making.

## Conclusions

Frailty demonstrated more consistent and clinically meaningful associations than chronological age across multiple pain characteristics, although effect sizes were modest. Incorporating frailty assessment–an indirect measure of biological age–into chronic pain characterization provides a more nuanced understanding of vulnerability and may facilitate earlier, individualized management strategies. This approach could improve pain control, reduce adverse outcomes, and help disrupt the frailty-pain cycle, while avoiding diagnostic simplifications often driven by chronological age alone. Integrating frailty-informed frameworks into clinical practice and guideline development may therefore enhance both precision and equity in pain care for older adults.

## Supplementary Information

Below is the link to the electronic supplementary material.

Supplementary Material 1

## Data Availability

The datasets generated and analyzed during the present study are available from the corresponding author upon reasonable request.

## References

[1] Treede RD, Rief W, Barke A et al (2018) Chronic pain as a symptom or a disease: the IASP classification of chronic pain for the international classification of diseases (ICD-11). Pain 160(1):19–27. 10.1097/j.pain.0000000000001384 10.1097/j.pain.0000000000001384 30586067

[2] Karp JF, Shega JW, Morone NE, Weiner DK (2008) Advances in Understanding the mechanisms and management of persistent pain in older adults. Br J Anaesth 101(1):111–120. 10.1093/bja/aen090 18487247 10.1093/bja/aen090 PMC2841779

[3] Domenichiello AF, Ramsden CE (2019) The silent epidemic of chronic pain in older adults. Prog Neuropsychopharmacol Biol Psychiatry 93:284–290. 10.1016/j.pnpbp.2019.04.006 31004724 10.1016/j.pnpbp.2019.04.006 PMC6538291

[4] Imai R, Imaoka M, Nakao H et al (2020) Association between chronic pain and pre-frailty in Japanese community-dwelling older adults: A cross-sectional study. PLoS ONE 15(8 August):1–13. 10.1371/journal.pone.0236111 10.1371/journal.pone.0236111 PMC7425941 32790685

[5] Thapa S, Shmerling RH, Bean JF, Cai Y, Leveille SG (2019) Chronic multisite pain: evaluation of a new geriatric syndrome. Aging Clin Exp Res 31(8):1129–1137. 10.1007/s40520-018-1061-3 30361952 10.1007/s40520-018-1061-3 PMC6483883

[6] Pitcher MH, Von Korff M, Bushnell MC, Porter L (2019) Prevalence and profile of high-impact chronic pain in the united States. J Pain 20(2):146–160. 10.1016/j.jpain.2018.07.006 30096445 10.1016/j.jpain.2018.07.006 PMC8822465

[7] Brown L, Young J, Clegg A, Heaven A (2015) Pain in older people with frailty. Rev Clin Gerontol 25(3):159–171. 10.1017/S0959259815000143

[8] Rastogi R, Meek BD (2013) Management of chronic pain in elderly, frail patients: finding a suitable, personalized method of control. Clin Interv Aging 8:37–46. 10.2147/CIA.S30165 23355774 10.2147/CIA.S30165 PMC3552607

[9] Eggermont LH, Leveille SG, Shi L et al (2014) Pain characteristics associated with the onset of disability in older adults: the maintenance of balance, independent living, intellect, and zest in the elderly Boston study. J Am Geriatr Soc 62(6):1007–1016. 10.1111/jgs.12848 24823985 10.1111/jgs.12848 PMC4057984

[10] Rundell SD, Patel KV, Krook MA et al (2019) Multisite pain among older adults with persistent back pain and its association with longitudinal outcomes. Pain Med 20(10):1898–1906. 10.1093/pm/pny270 30615144 10.1093/pm/pny270

[11] Rockwood K (2005) What would make a definition of frailty successful? Age Ageing 34(5):432–434. 10.1093/ageing/afi146 16107450 10.1093/ageing/afi146

[12] Fried LP, Tangen CM, Walston J et al (2001) Frailty in older adults: evidence for a phenotype. J Gerontol Biol Sci Med Sci 56(3):146–157. 10.1093/gerona/56.3.m146 10.1093/gerona/56.3.m146 11253156

[13] Dent E, Morley JE, Cruz-Jentoft AJ et al (2019) Physical frailty: ICFSR international clinical practice guidelines for identification and management. J Nutr Heal Aging 23(9):771–787. 10.1007/s12603-019-1273-z 10.1007/s12603-019-1273-z PMC6800406 31641726

[14] Mitnitski AB, Mogilner AJ, Rockwood K (2001) Accumulation of deficits as a proxy measure of aging. ScientificWorldJournal 1:323–336. 10.1100/tsw.2001.58 12806071 10.1100/tsw.2001.58 PMC6084020

[15] Searle SD, Mitnitski A, Gahbauer EA, Gill TM, Rockwood K (2008) A standard procedure for creating a frailty index. BMC Geriatr 8:1–10. 10.1186/1471-2318-8-24 18826625 10.1186/1471-2318-8-24 PMC2573877

[16] Theou O, Haviva C, Wallace L, Searle SD, Rockwood K (2023) How to construct a frailty index from an existing dataset in 10 steps. Age Ageing 52(12):1–7. 10.1093/ageing/afad221 10.1093/ageing/afad221 PMC10733590 38124255

[17] Rockwood K, Andrew M, Mitnitski A (2007) A comparison of two approaches to measuring frailty in elderly people. J Gerontol Biol Sci Med Sci 62(7):738–743. 10.1093/gerona/62.7.738 10.1093/gerona/62.7.738 17634321

[18] Theou O, Rockwood MRH, Mitnitski A, Rockwood K (2012) Disability and co-morbidity in relation to frailty: how much do they overlap? Arch Gerontol Geriatr 55(2):e1. 10.1016/j.archger.2012.03.001 22459318 10.1016/j.archger.2012.03.001

[19] Mitnitski AB, Song X, Rockwood K (2004) The Estimation of relative fitness and frailty in community-dwelling older adults using self-report data. J Gerontol Biol Sci Med Sci 59(6):627–632. 10.1093/gerona/59.6.m627 10.1093/gerona/59.6.m627 15215283

[20] Hoogendijk EO, Afilalo J, Ensrud KE, Kowal P, Onder G, Fried L (2019) Frailty: implications for clinical practice and public health. Lancet 394(10206):1365–1375. 10.1016/S0140-6736(19)31786-6 31609228 10.1016/S0140-6736(19)31786-6

[21] Mitnitski AB, Graham JE, Mogilner AJ, Rockwood K (2002) Frailty, fitness and late-life mortality in relation to chronological and biological age. BMC Geriatr 2:1–8. 10.1186/1471-2318-2-1 11897015 10.1186/1471-2318-2-1 PMC88955

[22] Diebel LW, Rockwood K (2021) Determination of biological age: geriatric assessment vs biological biomarkers. Curr Oncol Rep 23(9). 10.1007/s11912-021-01097-9 10.1007/s11912-021-01097-9 PMC8284182 34269912

[23] Theou O, Walston J, Rockwood K (2015) Operationalizing frailty using the frailty phenotype and deficit accumulaton approaches. Interdiscip Top Gerontol Geriatr 41:66–73. 10.1159/000381164 26301980 10.1159/000381164 PMC4886227

[24] Theou O, Rockwood K (2015) Comparison and clinical applications of the frailty phenotype and frailty index approaches. Interdiscip Top Gerontol Geriatr 41:74–84. 10.1159/000381166 26301981 10.1159/000381166

[25] Saraiva MD, Suzuki GS, Lin SM, de Andrade DC, Jacob-Filho W, Suemoto CK (2018) Persistent pain is a risk factor for frailty: A systematic review and meta-analysis from prospective longitudinal studies. Age Ageing 47(6):785–793. 10.1093/ageing/afy104 30052700 10.1093/ageing/afy104

[26] Lin T, Zhao Y, Xia X, Ge N, Yue J (2020) Association between frailty and chronic pain among older adults: A systematic review and meta-analysis. Eur Geriatr Med 11(6):945–959. 10.1007/s41999-020-00382-3 32808241 10.1007/s41999-020-00382-3

[27] Megale RZ, Ferreira ML, Ferreira PH et al (2018) Association between pain and the frailty phenotype in older men: longitudinal results from the concord health and ageing in men project (CHAMP). Age Ageing 47(3):381–387. 10.1093/ageing/afy012 29474508 10.1093/ageing/afy012

[28] Chiou JH, Liu LK, Lee WJ, Peng LN, Chen LK (2018) What factors mediate the inter-relationship between frailty and pain in cognitively and functionally sound older adults? A prospective longitudinal ageing cohort study in Taiwan. BMJ Open 8(2):1–9. 10.1136/bmjopen-2017-018716 10.1136/bmjopen-2017-018716 PMC5829604 29453297

[29] Veronese N, Maggi S, Trevisan C et al (2017) Pain increases the risk of developing frailty in older adults with osteoarthritis. Pain Med 18(3):414–427. 10.1093/pm/pnw163 27497322 10.1093/pm/pnw163

[30] Lohman MC, Whiteman KL, Greenberg RL, Bruce ML (2017) Incorporating persistent pain in phenotypic frailty measurement and prediction of adverse health outcomes. J Gerontol Biol Sci Med Sci 72(2):216–222. 10.1093/gerona/glw212 10.1093/gerona/glw212 PMC5233918 28087677

[31] Tian X, Wang C, Qiao X et al (2018) Association between pain and frailty among Chinese community-dwelling older adults: depression as a mediator and its interaction with pain. Pain 159(2):306–313. 10.1097/j.pain.0000000000001105 29140924 10.1097/j.pain.0000000000001105

[32] de Morais D, Terassi M, Inouye K, Luchesi BM, Pavarini SCI (2017) Chronic pain in elderly caregivers at different levels of frailty. Rev Gauch Enferm 37(4):e60700. 10.1590/1983-1447.2016.04.60700 10.1590/1983-1447.2016.04.60700 28198946

[33] Tse MMY (2016) Frailty, pain and psychological variables among older adults living in Hong Kong nursing homes: can we do better to address multimorbidities? J Psychiatr Ment Health Nurs 23(5):303–311. 10.1111/jpm.12303 27307261 10.1111/jpm.12303

[34] Coyle P, Sions J, Velasco T, Chicks G (2015) Older adults with chronic low back pain: A clinical population vulnerable to frailty? J Frailty Aging 4(4):1–3. 10.14283/jfa.2015.75 10.14283/jfa.2015.75 PMC4820763 27031017

[35] Misra D, Felson DT, Silliman RA et al (2015) Knee osteoarthritis and frailty: findings from the multicenter osteoarthritis study and osteoarthritis initiative. J Gerontol Biol Sci Med Sci 70(3):339–344. 10.1093/gerona/glu102 10.1093/gerona/glu102 PMC4351392 25063080

[36] Wise BL, Parimi N, Zhang Y et al (2014) Frailty and hip osteoarthritis in men in the MrOS cohort. J Gerontol Biol Sci Med Sci 69(5):602–608. 10.1093/gerona/glt126 10.1093/gerona/glt126 PMC3991147 24253535

[37] Livshits G, Lochlainn MN, Malkin I et al (2018) Shared genetic influence on frailty and chronic widespread pain: A study from TwinsUK. Age Ageing 47(1):119–125. 10.1093/ageing/afx122 28985290 10.1093/ageing/afx122 PMC5860041

[38] Hermsen L, Leone S, Smalbrugge M, Dekker J, van der Horst H (2014) Frequency, severity and determinants of functional limitations in older adults with joint pain and comorbidity: results of a cross-sectional study. Arch Gerontol Geriatr 59(1):98–106. 10.1016/j.archger.2014.02.006 24726180 10.1016/j.archger.2014.02.006

[39] Koponen M, Bell S, Karttunen N, Nykänen I, Desplenter F, Hartikainen S (2013) Analgesic use and frailty among community-dwelling older people: A population-based study. Drugs Aging 30(2):129–136. 10.1007/s40266-012-0046-8 23288603 10.1007/s40266-012-0046-8

[40] Weaver GD, Kuo YF, Raji MA et al (2009) Pain and disability in Mexican-American older adults. J Am Geriatr Soc 57(6):992–999. 10.1111/j.1532-5415.2009.02263.x 19453304 10.1111/j.1532-5415.2009.02263.x PMC2690616

[41] Ning H, Zhao Y, Liao L et al (2022) Impact of pain and psychosocial factors on frailty among older adults with physical functional limitations: A cross-sectional study. Pain Manag Nurs 23(3):338–344. 10.1016/j.pmn.2021.04.007 33994304 10.1016/j.pmn.2021.04.007

[42] Tsuji S, Shinmura K, Nagai K et al (2021) Low back pain is closely associated with frailty but not with sarcopenia: Cross-sectional study of rural Japanese community-dwelling older adults. Geriatr Gerontol Int 21(1):54–59. 10.1111/ggi.14100 33245209 10.1111/ggi.14100

[43] Otones Reyes P, García Perea E, Rico Blázquez M, Pedraz Marcos A (2020) Prevalence and correlates of frailty in community-dwelling-older adults with chronic pain: A cross-sectional study. Pain Manag Nurs 21(6):530–535. 10.1016/j.pmn.2020.05.009 32636062 10.1016/j.pmn.2020.05.009

[44] İlhan B, Bahat G, Erdoğan T, Kılıç C, Karan MA (2019) Chronic pain: prevalent and independently associated with frailty and female gender in geriatric outpatients. Eur Geriatr Med 10(6):931–937. 10.1007/s41999-019-00235-8 34652781 10.1007/s41999-019-00235-8

[45] Esses GJ, Liu X, Lin HM, Khelemsky Y, Deiner S (2019) Preoperative frailty and its association with postsurgical pain in an older patient cohort. Reg Anesth Pain Med 44(7):695–699. 10.1136/rapm-2018-100247 10.1136/rapm-2018-100247 31061107

[46] Nakai Y, Makizako H, Kiyama R, Tomioka K, Taniguchi Y, Kubozono T et al (2020) Association between chronic pain and physical frailty in community-dwelling older adults. Int J Environ Res Public Health 17(1):5–8. 10.3390/ijerph16081330 10.3390/ijerph17010175 PMC6982224 31881764

[47] Bindawas SM, Vennu V, Stubbs B (2018) Longitudinal relationship between knee pain status and incident frailty: data from the osteoarthritis initiative. Pain Med 19(11):2146–2153. 10.1093/pm/pnx296 29206993 10.1093/pm/pnx296 PMC6659024

[48] Ardoino I, Franchi C, Nobili A et al (2020) Pain and frailty in hospitalized older adults. Pain Ther 9(2):727–740. 10.1007/s40122-020-00202-3 33058084 10.1007/s40122-020-00202-3 PMC7648833

[49] Rodríguez-Sánchez I, García-Esquinas E, Mesas AE, Martín-Moreno JM, Rodríguez-Mañas L, Rodríguez-Artalejo F (2019) Frequency, intensity and localization of pain as risk factors for frailty in older adults. Age Ageing 48(1):74–80. 10.1093/ageing/afy163 30307470 10.1093/ageing/afy163

[50] Wade KF, Marshall A, Vanhoutte B, Wu FCW, O’Neill TW, Lee DM (2017) Does pain predict frailty in older men and women? Findings from the english longitudinal study of ageing (ELSA). J Gerontol Biol Sci Med Sci 72(3):403–409. 10.1093/gerona/glw226 10.1093/gerona/glw226 PMC5861874 27836906

[51] Wade KF, Lee DM, Mcbeth J et al (2016) Chronic widespread pain is associated with worsening frailty in European men. Age Ageing 45(2):268–274. 10.1093/ageing/afv170 26679698 10.1093/ageing/afv170 PMC4776622

[52] Coelho T, Paúl C, Gobbens RJ, Fernandes L (2017) Multidimensional frailty and pain in community dwelling elderly. Pain Med 18(4):693–701. 10.1111/pme.12746 25800906 10.1111/pme.12746

[53] Shega JW, Andrew M, Kotwal A (2013) Relationship between persistent pain and 5-year mortality: A population-based prospective cohort study. J Am Geriatr Soc 61(12):2135–2141. 10.1111/jgs.12554 24320761 10.1111/jgs.12554 PMC4140782

[54] Shega JW, Dale W, Andrew M (2012) Persistent pain and frailty: A case for homeostenosis. J Am Geriatr Soc 60(1):113–117. 10.1111/j.1532-5415.2011.03769.x 22150394 10.1111/j.1532-5415.2011.03769.x PMC3258356

[55] Shega JW, Andrew M, Hemmerich J et al (2012) The relationship of pain and cognitive impairment with social vulnerability-An analysis of the Canadian study of health and aging. Pain Med 13(2):190–197. 10.1111/j.1526-4637.2011.01309.x 22239723 10.1111/j.1526-4637.2011.01309.x

[56] Hirase T, Makizako H, Okubo Y, Lord SR, Inokuchi S, Okita M (2019) Chronic pain is independently associated with social frailty in community-dwelling older adults. Geriatr Gerontol Int 19(11):1153–1156. 10.1111/ggi.13785 31646711 10.1111/ggi.13785

[57] Hirase T, Kataoka H, Nakano J, Inokuchi S, Sakamoto J, Okita M (2018) Impact of frailty on chronic pain, activities of daily living and physical activity in community-dwelling older adults: A cross-sectional study. Geriatr Gerontol Int 18(7):1079–1084. 10.1111/ggi.13314 29582534 10.1111/ggi.13314

[58] Cabrero-García J, Rico-Juan JR, Oliver-Roig A (2022) Does the global activity limitation indicator measure participation restriction? Data from the European Health and Social Integration Survey in Spain. Qual Life Res 31(5):1335–44. 10.1007/s11136-021-03057-z 10.1007/s11136-021-03057-z PMC9023392 34882281

