# Supplementary material for: From age to frailty: redefining chronic pain characterization
Source: Aging Clin Exp Res. 2025 Dec 2;38(1):25. doi: 10.1007/s40520-025-03273-4 (PMC12774935; doi:10.1007/s40520-025-03273-4)
Supplement: Supplementary file 1 — Supplementary Material 1 [file 40520_2025_3273_MOESM1_ESM.docx]

**Supplementary Material**

**Note to the reader**

This supplementary material complements the main manuscripts by detailing the variables included in the Frailty Index (FI), the distribution of FI scores in the analytical sample, and graphical representations of selected associations between frailty and chronic pain characteristics. The included figures were selected for their illustrative value in depicting distributional trends and gradient effects across frailty levels. These materials enhance interpretability and transparency while avoiding redundancy with the main text.

**Appendix 1: Recruitment flow and analytical sample composition (n=455)**

| **Recruitment step** | **n (%) baseline** | **Notes** |
| --- | --- | --- |
| Patients initially screened | 1646 (100%) | Consecutive recruitment at two primary care centers including both community-dwelling and institutionalized participants |
| Met IASP chronic pain criteria | 713 (43.3%) | Includes repeated and institutionalized |
| Institutionalized participants | 37 (8.1%) | Not excluded per se; retained in the analytical sample |
| Final analytical sample (interviewed) | 455 (63.8% of chronic pain cases) | Participants completing the structured assessment |

**Appendix 2: 31 frailty index variables**

| **Comorbidities or chronic conditions (n=22)** | **Mental health (n=4)** | **Physical function and disability (n=3)** | **Social health (n=1)** | **Self-rated health (n=1)** |
| --- | --- | --- | --- | --- |
| Arterial hypertension  Diabetes mellitus  Anxiety  Depression  Sleep disorders  Other psychiatric disorders  Sequelae of stroke  Adverse effects of chemotherapy and/or radiotherapy  Surgical interventions  Herpes zoster  Central nervous system infections  Rheumatoid arthritis  Osteoarthritis  Fractures  Osteoporosis  Other arthritis  Trauma surgery  Tendinopathy  Spinal canal stenosis  Other musculoskeletal conditions  Total number of drugs  Other comorbidities | Do less carefully during the past 4 weeks due to emotional problem (SF-12)  Time feel calm and peaceful during the past 4 weeks (SF-12)  Time a lot of energy during the last 4 weeks (SF-12)  Time feeling discouraged and sad during the last 4 weeks (SF-12) | Moderate effort limitation (SF-12)  Go up several floors by stairs limitation (SF-12)  Stop doing some tasks due to physical health (SF-12) | Social limitation during the past 4 weeks due to physical health or emotional problems (SF-12) | General health (SF-12) |

Although “Rheumatoid arthritis” and “other arthritis” were not associated with age in our clinical dataset, they were included in the Frailty Index (FI) due to their established inclusion in other FIs and their well-documented relationship with age.

**Appendix 3: Frailty Index characteristics**

| **Frailty Index Categories** | |  | **Weighted frequency** | |
| --- | --- | --- | --- | --- |
|  | |  | **Absolute** | **Relative (%) (95% CI)** |
| 0-0.1 | **Non-Frail** |  | 53 | 11.7% (8.9%-14.8%) |
| 0.11-0.2 | **Very Mildly Frail** |  | 94 | 20.7% (17.1%-24.6%) |
| 0.21-0.3 | **Mildly Frail** |  | 142 | 31.1% (27.1%-35.6%) |
| 0.31-0.4 | **Moderately Frail** |  | 76 | 16.7% (13.5%-20.3%) |
| 0.41+ | **Severely Frail** |  | 90 | 19.8% (16.3%-23.6%) |

**
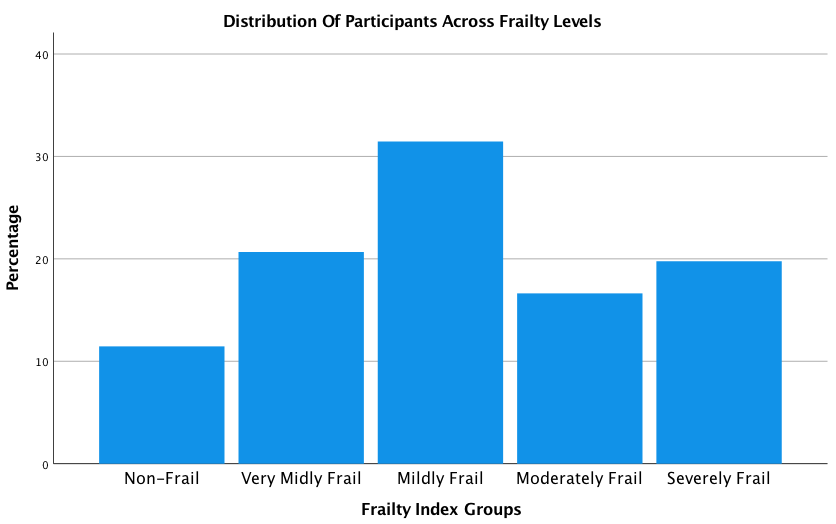
**


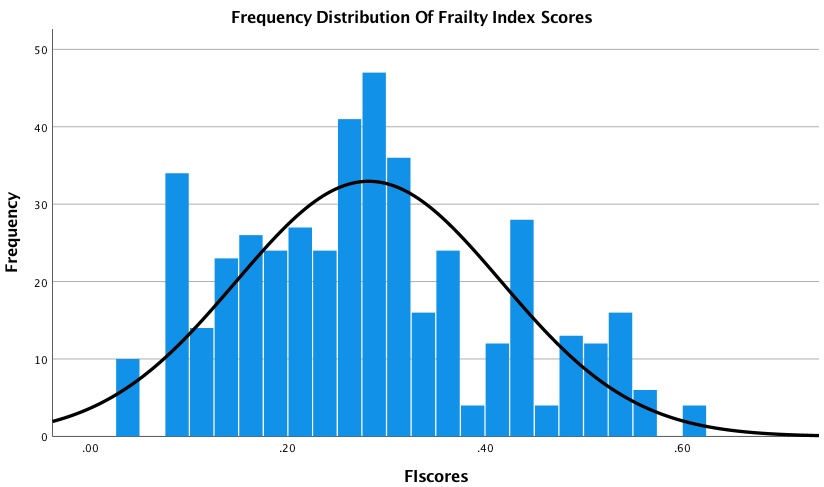


Frailty index score range from 0.04 to 0.62 / Mean 0.28 / SD 0.13 / 99^th^ percentile 0.57

**
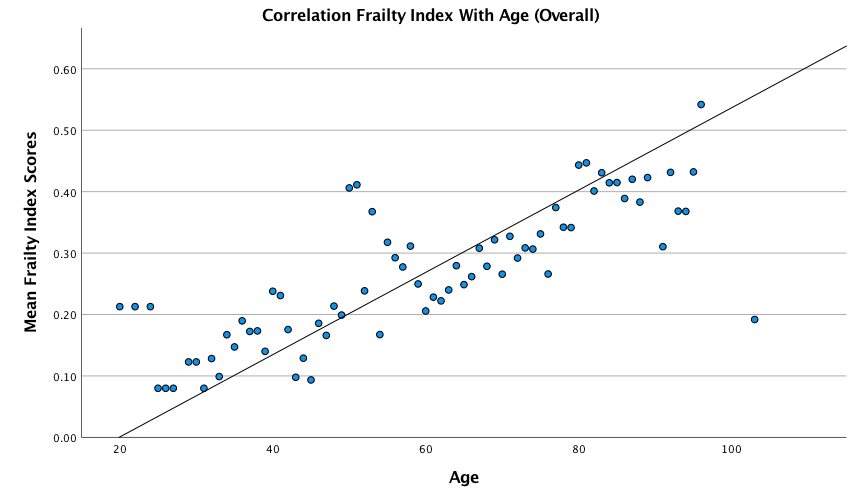
**

| **Variables** | **Correlation coefficient** | **Significance** | **Sample size (N)** |
| --- | --- | --- | --- |
| Age-FIscore | *Pearson (r)*=0.625 | p<0.001 | 455 |


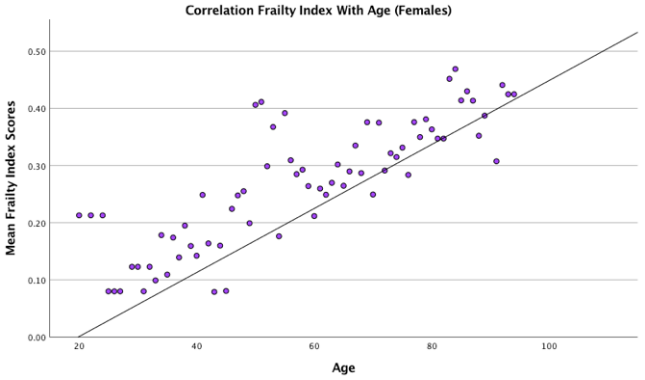

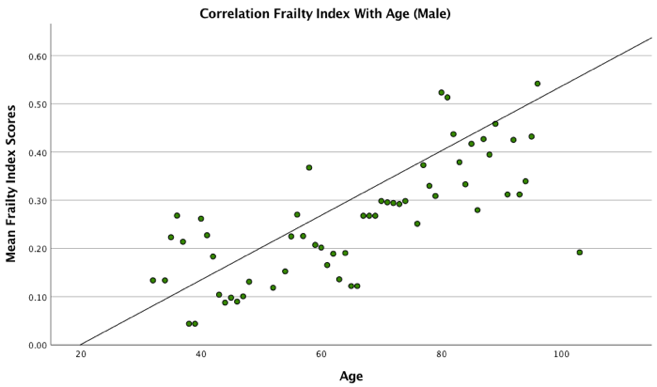


| **Variables** | Age-FIscore in women | Age-FIscore in men | |
| --- | --- | --- | --- |
| **Correlation** | *Pearson (r)*=0.632 | *Pearson (r)*=0.648 | |
| **Significance** | *p < 0.001* | p < 0.001 |  |
| **Sample size (N)** | *269* | 176 |  |
|  | 455 | | |

| **Variables** | **ANOVA** | **Significance** | **Sample size (N)** |
| --- | --- | --- | --- |
| sex-FIscore | Means:   - Mean females: 0.285 - Mean males: 0.277 | p = 0.520 | 455 |

**Appendix 4: Pain characteristics across frailty levels**


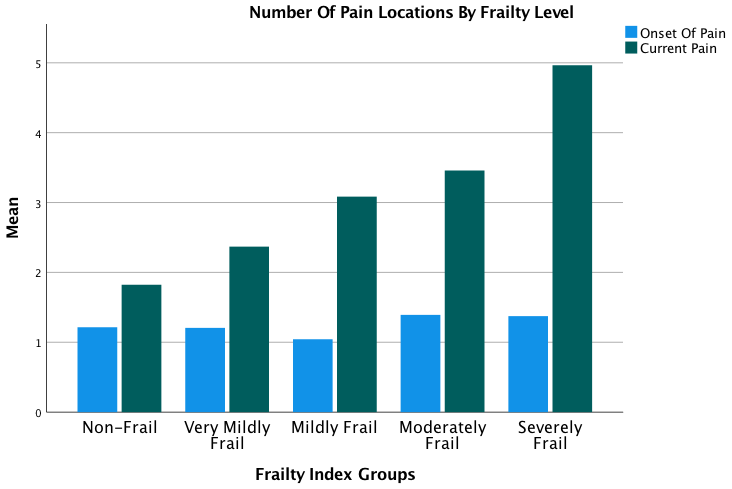


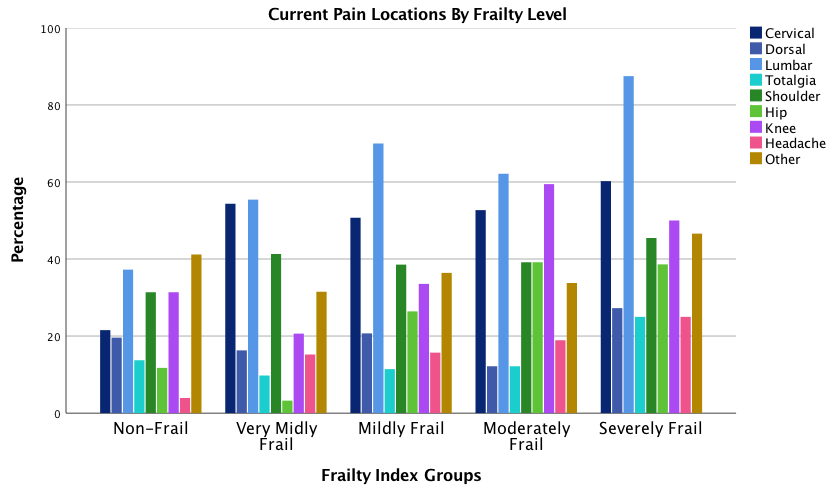


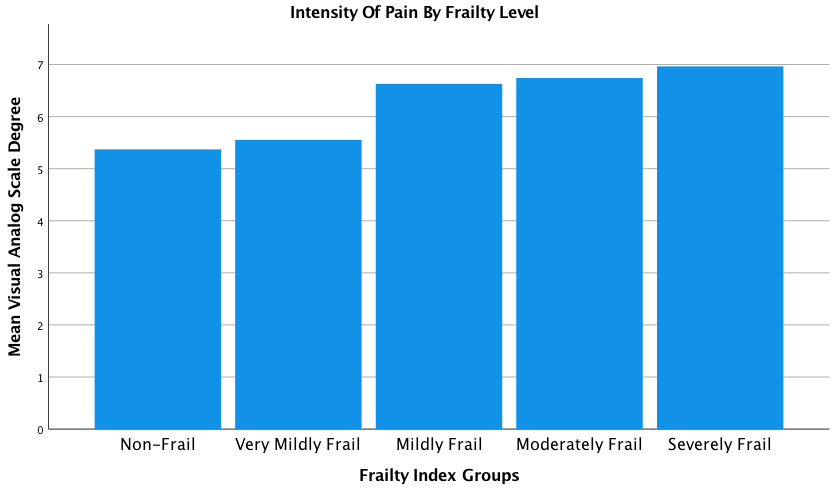


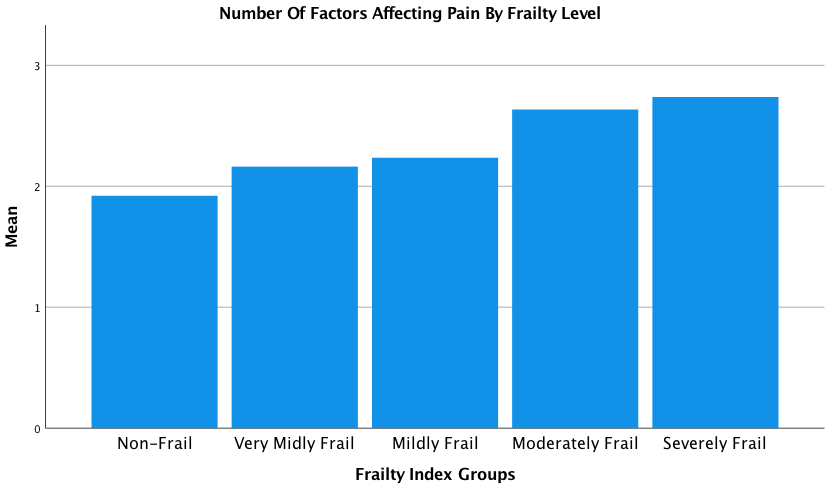


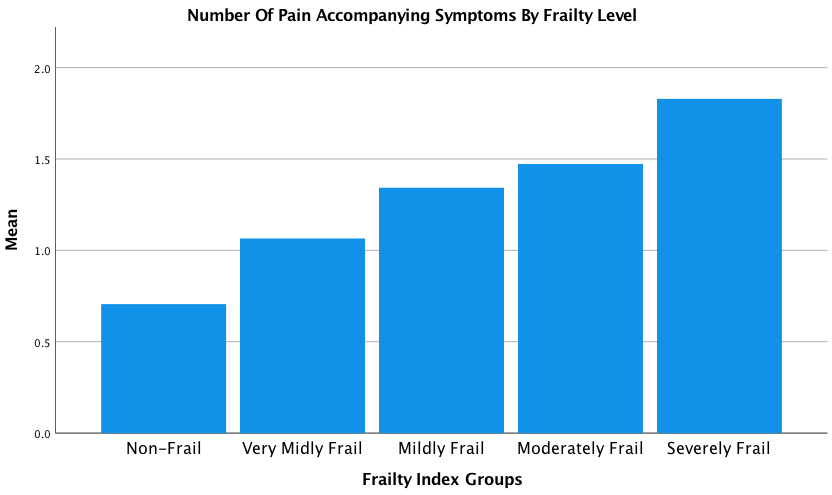


**Appendix 5: Associations between chronic pain characteristics and sex, age and frailty**

**Table 3a.** Continuous outcomes: Unstandardized β (95% confidence intervals) and semipartial correlations (variance explained)

| **Domain** | **Pain characteristics** | **Sex** | | | | | **Age** | | **Frailty** | | |
| --- | --- | --- | --- | --- | --- | --- | --- | --- | --- | --- | --- |
| **Duration of pain (months)** | Since the onset of pain | -13.663  (-42.701-15.375) | -0.03 (0.10%) | | | | **5.996 (5.019-6.973)** | **0.422 (17.81%)** | **2.472 (1.139-3.806)** | **0.128 (1.64%)** | |
|  | Between the onset of pain and the etiological diagnosis | -21.745  (-44.555-1.066) | -0.076 (0.58%) | | | | **3.908 (3.132-4.685)** | **0.402 (16.16%)** | **1.389 (0.342-2.436)** | **0.106 (1.12%)** | |
|  | Between the onset of pain and the first prescription (pharmacological and non-pharmacological) | **-33.050**  **(-50.196 to -15.903)** | **-0.144 (2.07%)** | | | | **2.547 (1.973-3.121)** | **0.332 (11.02%)** | **1.471 (0.686-2.256)** | **0.140 (1.96%)** | |
|  | Between the onset of pain and its control | -25.887  (-52.514-0.739) | -0.095 (0.90%) | | | | **2.243 (1.346-3.139)** | **0.244 (5.95%)** | **3.542 (2.238-4.846)** | **0.265 (7.02%)** | |
| **Pain locations** | Number of pain locations at onset of pain | **0.269 (0.172-0.366)** | **0.250 (6.25%)** | | | | 0.002  (-0.002-0.005) | 0.047 (0.22%) | 0.003  (-0.002-0.007) | 0.058 (0.34%) | |
|  | Number of current pain locations | **0.376 (0.065-0.687)** | **0.095 (0.90%)** | | | | **0.022 (0.012-0.033)** | **0.165 (2.72%)** | **0.053 (0.039-0.068)** | **0.293 (8.58%)** | |
| **Factors affecting pain** | Number of factors affecting pain | **0.178 (0.035-0.321)** | **0.108 (1.17%)** | | | | **0.007 (0.002-0.012)** | **0.122 (1.49%)** | **0.015 (0.008-0.021)** | **0.192 (3.69%)** | |
| **Accompanying symptoms** | Number of pain accompanying symptoms | **0.256 (0.060-0.452)** | **0.105 (1.10%)** | | | | **-0.027**  **(-0.034 to -0.021)** | **-0.333 (11.09%)** | **0.052 (0.043-0.061)** | **0.463 (21.44%)** | |
| **Other characteristics** | Frequency of pain (days/week) | **0.190 (0.093-0.287)** | | **0.178 (3.17%)** | | | 0.001  (-0.002-0.004) | 0.028 (0.008%) | **0.005 (0.001-0.010)** | | **0.104 (1.08%)** |
|  | Duration of pain per day (hours) | **1.791 (0.703-2.879)** | | | **0.149 (2.22%)** | | **-0.055**  **(-0.092 to -0.019)** | **-0.136 (1.85%)** | **0.098 (0.048-0.148)** | | **0.177 (3.13%)** |
|  | Intensity of pain | **0.426 (0.146-0.706)** | | | | **0.131 (1.72%)** | 0.009 (0.001-0.019) | 0.082 (0.67%) | **0.035 (0.022-0.047)** | | **0.230 (5.29%)** |

**Bolded values** indicate statistical significance (p<0.05)

**Table 3b.** Categorical outcomes: Odds ratios with 95% confidence intervals

| **Domain of pain** | **Pain variables** | **% outcome** | **Sex** | **Age** | **Frailty** |
| --- | --- | --- | --- | --- | --- |
| **Pain locations** | Cervical location (current) | 224 (50.3%) | **4.191 (2.739-6.413)** | 1.003 (0.989-1.017) | **1.022 (1.002-1.042)** |
|  | Dorsal location (current) | 87 (19.6%) | 1.446 (0.860-2.433) | **0.977 (0.961-0.994)** | **1.033 (1.009-1.057)** |
|  | Lumbar location (current) | 291 (65.4%) | 0.854 (0.552-1.321) | 1.012 (0.997-1.026) | **1.045 (1.023-1.068)** |
|  | Total location (current) | 63 (14.2%) | **2.037 (1.102-3.768)** | 1.008 (0.989-1.028) | 1.021 (0.996-1.047) |
|  | One shoulder location (current) | 177 (39.8%) | **1.653 (1.093-2.500)** | **1.026 (1.011-1.040)** | 0.983 (0.964-1.002) |
|  | Both shoulders location (current) | 30 (7.0%) | 1.288 (0.578-2.871) | 1.019 (0.991-1.048) | 1.018 (0.984-1.054) |
|  | One hip location (current) | 109 (24.5%) | **1.851 (1.122-3.053)** | **1.024 (1.007-1.041)** | **1.035 (1.013-1.057)** |
|  | One knee location (current) | 170, (38.2%) | 1.330 (0.857-2.064) | **1.049 (1.033-1.066)** | 1.001 (0.981-1.021) |
|  | Both knees location (current) | 31 (7.0%) | 0.788 (0.334-1.860) | **1.089 (1.040-1.142)** | **1.064 (1.024-1.105)** |
|  | Headache location (current) | 74 (16.6%) | **1.914 (1.070-3.423)** | 0.985 (0.967-1.003) | **1.047 (1.021-1.073)** |
|  | Other locations (current) | 167 (37.5%) | **0.601 (0.401-0.901)** | 0.997 (0.983-1.010) | 1.012 (0.993-1.031) |
| **Factors affecting pain** | Movement affecting pain | 397 (89.2%) | 1.210 (0.633-2.314) | **1.024 (1.001-1.048)** | **1.038 (1.003-1.074)** |
|  | Temperature affecting pain | 307 (69.0%) | 1.108 (0.718-1.711) | 1.005 (0.990-1.019) | **1.038 (1.017-1.061)** |
|  | Medication affecting pain | 338 (76.0%) | **2.272 (1.408-3.665)** | **1.018 (1.002-1.035)** | **1.037 (1.013-1.063)** |
| **Pain accompanying symptoms** | Nausea as a pain accompanying symptom | 56 (12.6%) | 1.699 (0.882-3.274) | **0.965 (0.945-0.985)** | **1.042 (1.014-1.071)** |
|  | Asthenia as a pain accompanying symptom | 196 (44.0%) | **2.173 (1.390-3.395)** | **0.961 (0.945-0.976)** | **1.099 (1.074-1.126)** |
|  | Anorexia as a pain accompanying symptom | 75 (16.9%) | **2.027 (1.115-3.684)** | 0.994 (0.975-1.013) | **1.072 (1.045-1.100)** |
|  | Sleep disturbance as a pain accompanying symptom | 266 (59.8%) | 1.259 (0.816-1.943) | **0.938 (0.921-0.955)** | **1.094 (1.068-1.121)** |
| **Other characteristics**  **of pain** | Type of pain (mechanical, neuropathic, mixed) | Mechanical 180 (40.5%)  Neuropathic  111 (24.9%)  Mixed 154 (34.6%) | 1.040 (0.681-1.589) | **1.015 (1.000-1.029)** | **1.033 (1.013-1.053)** |
|  | Course of pain (intermittent, continuous) | Intermittent 308 (69.2%)  Continuous 137 (30.8%) | **0.568 (0.360-0.896)** | 1.012 (0.997-1.028) | **0.969 (0.949-0.989)** |
|  | Social limitation due to pain | Not at all, quite a bit and neutral  235 (52.8%)  Quite a bit and a lot  210 (47.2%) | **0.372 (0.241-0.574)** | **1.035 (1.019-1.051)** | **0.920 (0.899-0.941)** |

**Bolded values** indicate statistical significance.

Note: “Both hips location” was excluded from multivariable regression analyses due to low event count (< 30 cases, 5.6%), which may lead to unstable estimates. However, adjusted logistic regression controlling for age, sex, and frailty yielded the following: age OR 1.011 (95% CI 0.977-1.046), sex OR 1.101 (95% CI 1.055-1.149), and frailty OR 3.201 (95% CI 1.077-9.518). Results should be interpreted with caution due to limited statistical power.
